# HIRSUTISM IN KASHMIR: AN ETIOLOGICAL STUDY

**DOI:** 10.4103/0019-5154.48997

**Published:** 2009

**Authors:** Qazi Masood Ahmad, Iffat Hassan Shah, Farah Sameem, Qurat-ul-ain Kamili, Javeed Sultan

**Affiliations:** *From the Department of Dermatology, STD and Leprosy, Government Medical College, Srinagar, Kashmir, India*

**Keywords:** *Ferriman-Gallwey scoring*, *hirsutism*, *polycystic ovaries*

## Abstract

**Background:**

Hirsutism refers to the presence of terminal hairs at the body sites under androgenic control. Various factors, including genetic makeup and hormonal status, influence the rate and pattern of hair growth at these sites.

**Purpose:**

To study the pattern of hirsutism in Kashmir.

**Materials and Methods:**

Thirty five consecutive patients of hirsutism were included in the study. After detailed history taking, physical examination and relevant investigations, scoring of hirsutism was done using the Ferriman Gallwey (FG) scoring system.

**Findings:**

The FG score ranged from 10-34. Twenty patients had associated menstrual abnormalities. Polycystic ovarian syndrome (PCOS) was diagnosed in four patients, hypothyroidism in two and congenital adrenal hyperplasia (CAH) in one. The rest of the patients had idiopathic hirsutism.

**Conclusion:**

Idiopathic hirsutism was the most common category, whilst PCOS, hypothyroidism and CAH were also seen.

## Introduction

Hirsutism refers to the presence of coarse terminal hair in a female at sites under androgenic control, such as the face, chest, abdomen and the inner aspect of thighs.[1] The rate, pattern and distribution of hair growth at these sites is influenced by various factors including an individual's genetic makeup and hormonal status.[2] A disturbance in the complex interaction between these factors can lead to a male pattern of hair growth in a female. Hirsutism may be idiopathic, i.e. secondary to increased responsiveness of the hair follicles to normal circulating levels of androgens, or it may result from an excess of androgens and other hormones. The source of the excess androgens may be either the ovaries, the adrenals or increased peripheral conversion of weak androgenic hormones to more potent ones.[2,3]

## Materials and Methods

### Study Population

The study was conducted in the Department of Dermatology, STD and Leprosy, SMHS Hospital, an associate hospital of the Government Medical College, Srinagar.

A total of thirty five females presenting with hirsutism at the out patients department of the hospital were included in the study. All the cases were recorded on a standard proforma, after taking informed consent.

### Clinical evaluation

Detailed medical history taking and physical examination of the patient were carried out in all the cases. Specific points recorded in the history included: age of onset, duration, rate of progression of the disease, marital status of the patient and the presence of symptoms of virilization (i.e. deepening of voice, thinning of scalp hair, increased muscularity, increased sebum production, acne, decreased breast size, and oligomenorrhoea). Family history of the disease and history of psychiatric illness and its treatment were also specifically sought for.

A thorough physical examination with specific emphasis on signs of virilization (including frontal baldness, loss of female body contours, increased muscularity, acne, clitoromegaly, and atrophy of breast) was done in all the patients.

### Assessment of degree of hirsutim

This was done using the Ferriman and Gallwey (FG) scoring system.

### Investigations

The levels of serum testosterone, sex hormone binding globulins, luteinizing hormone (LH), follicle stimulating hormone (FSH), prolactin, cortisol and abdominal and pelvic ultrasound for adrenals and ovaries were carried out in all the patients. Thyroid function tests, growth hormone level, dehydroepiandrosterone sulfate (DHEAS), 17-hydroxyprogesterone, X-ray skull, and CT (computerized tomography) of the abdomen were carried out in selected patients only.

## Results

Thirty five patients of hirsutism were enrolled in the study. The age range varied from 16 to 40 years, most of them being college-going students. All of them were native Kashmiris. A majority of the patients belonged to the age group of 21-25 years (41.7%), followed by 16-20 years (32.78%); 26-30 years (20.27%); 31-35 years (3.7%) and between 36 and 40 years (2.0%). The age of the onset of disease ranged from 10 to 24 years. The duration of hirsutism ranged from one to 15 years. Six (17.14 %) females were married, with two (5.7%) of them suffering from infertility. The severity of hirsutism, according to the Ferriman Gallwey score, ranged from 10 to 34, with twenty four (68.57%) patients having a score of more than 12. The face was the most common site, while the chest and abdomen were the next most common sites.

Twenty patients had associated menstrual abnormalities in the form of oligomenorrhea in twelve and menorrhagia in eight. Twenty patients had acne of varying grades. Twelve patients showed signs of frontal recession of the hair line, in an androgenic pattern. Eight patients were obese, on the basis of body mass index. Secondary sexual characteristics were well developed in all the patients, except one. Striae distensae were seen in eight patients. Thyroid symptoms suggestive of hypothyroidism were seen in two patients. A positive family history was obtained in nine (25.7%) patients, of whom five had a history in siblings while the other four had a history in maternal cousins. One of the patients was hirsute, with an FG score of more than 32, and with a history of amenorrhea, deepening of voice, clitoromegaly, muscular physique and short stature, with precocious puberty and childhood dehydration in male sibling. She was referred for complete endocrinological assessment, which revealed a diagnosis of congenital adrenal hyperplasia.

Serum testosterone levels were raised in two patients, one of whom had PCOS and the other had Idiopathic hirsutism. The LH/FSH ratio was elevated (>2) in four patients. All of them had PCOS. Serum Prolactin was increased in eight patients. Of these, two patients had hypothyroidism, three patients had PCOS, and three had Idiopathic hirsutism. One patient had elevated 17-hydroxyprogesterone levels and DHEAS. This patient was diagnosed as having congenital adrenal hyperplasia.

### Imaging techniques

Ultrasonographic findings of the patients are shown in the Table 1, which shows polycystic ovaries as the major ultrasonographic abnormality. In the case of one patient, a CT of the abdomen showed bilateral hyperplasia of the adrenals.

**Table 1:** Ultrasonographic findings of patients (*n* = 35)

Findings	*n* (%)
1. Normal	30 (85.71)
2. Polycystic ovaries	4 (11.43)
Unilateral cysts	2 (5.70)
Bilateral cysts	1 (2.86)
Bilaterally enlarged ovaries with multiple cysts	1 (2.86)
3. Adrenal pathology on ultrasound	1 (2.86)

### Causes of hirsutism

In our study, the commonest cause of hirsutism was Idiopathic hirsutism (80%). This was followed by PCOS, which was found in 11.43%. Other less frequent causes identified were hypothyroidism (5.70%) and congenital adrenal hyperplasia (2.86%).

## Discussion

Hirsutism is a common endocrine disorder responsible for a lot of anxiety amongst young women. Its spectrum varies from mild to severe; the severity being assessed by a semi-objective scoring system – the Ferriman and Gallwey Score.[4]

About five percent of women in the reproductive age group in the general population are hirsute, while about 25% of normal young women have some terminal hair in the face, areola or lower abdomen. The growth of terminal hair in male pattern is determined by the androgens and the intrinsic potential of the hair follicles to respond to the hormonal changes. The sensitivity of the hair follicle to androgens is largely governed by the alpha reductase activity in the skin, which is responsible for the conversion of testosterone to dihydrotestosterone. The severity of hirsutism does not correlate well with the level of androgens, because the response of the androgen dependent hair follicle varies considerably within and between individuals. The source of testosterone in a female is the ovaries and the adrenals. Androgen dependent hirsutism may be caused by disorders affecting the adrenals or ovaries, exogenous administration of androgens or a combination of these factors. Approximately half the women with mild hirsutism (FG score of 8-15) have idiopathic hirsutism. The most common identifiable cause of hyperandrogenic hirsutism appears to be polycystic ovarian syndrome (Stein Leventhal syndrome).[2–5]

Idiopathic hirsutism and Polycystic Ovary Syndrome account for 95% of the cases. Adrenal causes include congenital adrenal hyperplasia, Cushing's syndrome and androgen secreting adrenal tumors. Rarely, androgen secreting ovarian tumors, hyperprolactinemia, acromegaly, thyroid dysfunction, exogenous androgen intake in the form of anabolic steroids, oral contraceptives and hormone replacement therapy may be causative.[6,7] An increased incidence of hirsutism has been seen in relatives of hirsute women. This positive family history is seen both in cases of Idiopathic hirsutism as well as in cases of hirsutism, due to the underlying disorder.[8] Stress has also been implicated as an etiological factor for hirsutism. It is believed to be both a cause and a result of hirsutism.[9]

In our study, a majority of the patients were young. Hirsutism in this age group is generally of benign nature. A positive family history was obtained in 25.7% of the patients. The most common cause of hirsutism among our patients was idiopathic hirsutism (80%), followed by Polycystic Ovary Syndrome (11.43%). Hypothyroidism was seen in two patients, while congenital adrenal hyperplasia was seen in one patient.

After endocrinology assessment, these patients were started on appropriate medicines, including spironolactone, metformin, cyproterone acetate and topical eflornithine, amongst others. They were advised to undergo various hair removal procedures, including electrolysis and Laser hair removal. They continue to be on regular follow-up.
